# Editorial: Material and Structural Designs for Metal Ion Energy Storage Devices

**DOI:** 10.3389/fchem.2022.952440

**Published:** 2022-08-08

**Authors:** Fengkai Zuo, Yu Ding, Jun Zhang, Pan Xiong, Ping Wu, Hongsen Li

**Affiliations:** ^1^ Center for Marine Observation and Communications, College of Physics, Qingdao University, Qingdao, China; ^2^ College of Engineering and Applied Sciences, Nanjing University, Nanjing, China; ^3^ College of Materials Science and Engineering, Zhejiang University of Technology, Hangzhou, China; ^4^ Key Laboratory for Soft Chemistry and Functional Materials of Ministry Education, School of Chemistry and Chemical Engineering, Nanjing University of Science and Technology, Nanjing, China; ^5^ Jiangsu Key Laboratory of New Power Batteries, School of Chemistry and Materials Science, Nanjing Normal University, Nanjing, China

**Keywords:** electrode materials, structural design, metal-ion energy storage device, high energy densities, electrochemical performance

Driven by the increasing demand for portable electronics, grid-scale storage, and electric vehicles, intensive research on electrochemical energy storage (EES) devices with high performance that are cost-efficient and environmentally friendly is at the forefront of energy science and technology. Rechargeable metal-ion energy storage devices are considered to be promising candidates for sustainable large smart grids and renewable electrochemical energy storage technologies, owing to their high specific energy density, affordable cost, and long charge/discharge cycle life. The electrochemical properties and performance of these devices are intimately dependent on the physicochemical nature of their electrode materials, the critical component of an energy storage system, and the main redox species for electrochemical reactions. Further innovations and breakthroughs in electrode materials, rather than incremental changes are key to a new generation of energy storage devices. For these reasons, researchers have been highly motivated toward improving energy storage performance, exploring the fundamental properties of novel electrode materials with well-designed structures. Hence, this Research Topic of *Material and Structural Designs for Metal Ion Energy Storage Devices* focuses on the design of rational materials in different metal-ion-based energy storage devices.

In this Research Topic, representative types of materials design strategies are discussed in detail to provide reasonable solutions to compound problems and enable competitive performance for various real metal ion energy storage devices. They range from Lithium-ion batteries (LIBs) (Yu et al.; Ren et al.; Dong et al.; Wang et al.) to Sodium/Potassium ion batteries (Liu et al.) and solid-state Lithium batteries (Wang et al.; Lin et al.), and lithium-sulfur/selenium batteries (Lu et al.; Feng et al.). In addition to the 10 original research articles published here, our Research Topic also contains three review articles. Xu et al. review recent research progress in flexible rechargeable Zinc batteries. They discuss the distinction between different cathode, anode materials, and electrolytes, and in doing so, introduce the differences in preparation methods of electrode materials and their corresponding effect on flexible Zinc batteries. Li et al. provide a comprehensive overview of recent research on conversion-type thermal battery cathode materials. They generalize the preparation and characterization of numerous cathode materials and the performance testing of thermal cells. After that, the electrochemical behavior, properties, and problems occurring at the argyrodite solid-state electrolyte (SSE)/Li metal anode interface are summarized by Pang et al., who discuss strategies to stabilize interfaces and resolve interface problems in recent years and conclude the review with a brief future outlook for argyrodite SSEs. Their review papers highlight that this Research Topic is far from over and conclude by discussing fundamental issues and recommendations for future research trends.

Yu et al. provide a reliable route for the preparation of bimetal oxide materials, tailoring a core-shell structure for developing high-performance energy storage devices. They successfully obtained a core-shell ZnSnO_3_@ nitrogen-doped carbon (ZSO@NC) nanocomposite by *in-situ* polymerization of ZnSnO_3_ with polypyrrole (PPy) under ice bath conditions, finding it exhibits excellent electrochemical performance for lithium storage thanks to the unique compact structure.

Also exploring the field of LIBs, Wang et al. investigated the energy storage performance of the CuO/Cu_2_O/Cu nanocomposites and illustrate the advantages of multi-component synergy. Qin et al. demonstrated that the large-scale synthesis of NiS as an anode material for LIBs is promising due to its superior electrochemical performance and facile preparation method. Ren et al. indicated that the amorphous cobalt sulfide electrodes, with more structure defects, isotropy, and numerous grain boundaries, exhibit remarkable electrochemical performance. Dong et al. fabricated the well-designed hierarchical WO_3_ agglomerates and evaluated them as anode material of LIBs, which displays excellent potential for practical application in the field of high-energy-power LIBs.

Another work further transitions to other alkali metal ion battery systems, Liu et al. exploited a zeolitic imidazolate framework (ZIF)-derived hollow structures CoS/C for alkali ion (Li, Na, and K) battery anodes. There is also some work focusing on Solid-state electrolyte (SSE) lithium-ion batteries. Wang et al. utilized the electrospinning technique to prepare a three-dimensional Li_6.4_La_3_Zr_1.4_Ta_0.6_O_12_-Poly (Vinylidene Fluoride-Hexafluoropropylene) Gel Polymer Electrolyte, a potential candidate for safer and more stable solid-state lithium batteries. Lin et al. also gave an overview and discussed the effect of Si^4+^ concentration adjustment and elemental doping in obviously enhancing the ionic conductivity of tetrahedron Li_10.35_Si_1.35_P_1.65_S_12_. Their strategy can be easily extended to types of sulfide SSEs, thereby opening up previously unexplored opportunities in developing high-safety, high-performance, and long-life solid-state energy storage devices for practical applications.

Moving to the lithium-sulfur/selenium batteries, Lu et al. proposed a novel freestanding Se_1-x_S_x_ foamy cathodes assisted by supercritical CO_2_ technology for high-performance Li-S_1-x_S_x_ batteries. They not only provided a new strategy to prepare high-energy-density cathodes, but also a new method of fabricating free-standing cathodes for practical applications of next-generation recharge batteries. Feng et al. used the porous conductive reduced graphene oxide (rGO) loading polar CoS_2_ nanoparticles to mitigate the shuttle effect of polysulfides and speed up the electron/ion transfer. Thus, it was found that the Li-S cell with CoS_2_/rGO functional diaphragm exhibits enhanced electrochemical kinetics and high performance.

Although the papers collected here present significant insights, several problems and challenges need to be overcome in this field of research. Lower costs are one of the most prioritized considerations for the practical application of rechargeable energy storage devices, and further insights into the design of inexpensive electrode materials with promising electrochemical performance. It would also be significant to reveal the correlation between novel electrode designs and the charge storage mechanism, which will effectively guide the development of next-generation energy storage systems. Furthermore, the exploration of the electrolyte-electrode interface reaction mechanism creates room for corresponding electrode designs, which will advance the exploitation of cutting-edge characterization and analysis tools, while facilitating the development of new energy storage systems, especially those utilizing metal anodes or multivalent ions.

We thank all the authors for their meaningful work and would also like to thank all the reviewers for their insightful suggestions and constructive comments. It is hoped that this Research Topic will stimulate future research on the discovery and design of novel electrodes and drive the intensive ongoing development of high-energy-density rechargeable energy storage devices. We anticipate that these endeavors will pave the way for achieving green growth and stainable development.

